# A new clinical algorithm scoring for management of suspected foreign body aspiration in children

**DOI:** 10.1186/s12890-017-0406-6

**Published:** 2017-04-13

**Authors:** Ibrahim A. Janahi, Shabina Khan, Prem Chandra, Noora Al-Marri, Ammar Saadoon, Lolwa Al-Naimi, Maryam Al-Thani, William Greer

**Affiliations:** 1 Pediatric Pulmonology, Hamad Medical Corporation, P. O. Box 3050, Doha, Qatar; 2 Pediatrics, Hamad Medical Corporation, Doha, Qatar; 3 Medical Research Center, Hamad Medical Corporation, Doha, Qatar; 4 Clinical Epidemiology, Sidra Medical and Research Center, Doha, Qatar; 5 Clinical Pediatrics, Weill Cornell Medicine-Qatar, Doha, Qatar

**Keywords:** Foreign body, Pediatrics, Bronchoscopy, Airway obstruction, Predictors

## Abstract

**Background:**

Foreign Body Aspiration (FBA) is a serious problem in children and delays in diagnosis and management can be devastating. The history is often vague, with subtle physical and chest radiograph abnormalities. This study aims to determine the indications for bronchoscopy in children with suspected FBA and evaluate the key clinical and statistically significant predictors of FBA, based on the patients’ historical, physical and radiological findings at presentation.

**Methods:**

This is a retrospective observational study, including patients who were admitted between January 2001 to January 2011 with suspected FBA. Their presenting history, physical exam, radiological and bronchoscopic findings were analyzed.

**Results:**

Three hundred children with a mean age of 2.1 ± 1.7 years were included. In children with both abnormal physical and radiological findings, 47.2% had proven FBA. If either was abnormal, the likelihood reduced to 32–33.3%; if both were normal, only 7.4% had a FB. Witnessed choking (adjusted OR 2.1, 95% CI 1.03–4.3; *P* = 0.041), noisy breathing/stridor/dysphonia (adjusted OR 2.7, 95% CI 1.2–6.2; *P* = 0.015), new onset/recurrent /persistent wheeze (adjusted OR 4.6, 95% CI 1.8–11.8; *P* = 0.002), abnormal radiological findings (adjusted OR 4.0, 95% CI 1.9–8.5; *P* < 0.001), and unilateral reduced air entry (adjusted OR 2.9, 95% CI 1.5–5.5; *P* = 0.001) were significant predictors of FBA (*P* < 0.05). When three or more risk factors were present, the cumulative proportion of children with proven FBA increased significantly. The discriminative ability of the model was found to be good; the area under the ROC curve value was 0.76 (95% CI 0.70, 0.82). The predicted cutoff score derived using ROC analysis was found to co-relate well with known clinically significant predictors of FBA. This supports our algorithm and scoring system.

**Conclusions:**

A high index of suspicion is required in diagnosing airway FB. Our proposed clinical algorithm and scoring system hopes to empower physicians to accurately predict patients with a high likelihood of FBA.

## Background

Foreign Body Aspiration (FBA) is a serious common problem in children. Any delay in its diagnosis and management can result in devastating consequences [1–3]. FBA is quoted as the fifth cause of unintentional death among 1–3 year olds and the primary cause of unintentional death in babies under 12 months [4]. Physicians often struggle with the all too important, yet elusive, decision-“To bronch, or not to bronch” patients who present with suspected FBA. In most cases, the history is often vague, with only subtle (if any), physical and chest radiological abnormalities [5–10]. With this study, we aim to use our local experience in Qatar to retrospectively analyze bronchoscopically proven cases of FBA in an attempt to determine the statistically significant key clinical predictors of foreign body aspiration in children, based on the patient’s historical, physical and radiological findings at presentation.

## Methods

### Study design and participants

This is a retrospective observational study, including all patients aged 0 to 14 years, who were admitted to the pediatric department of Hamad Medical Corporation, Qatar between January 2001 to January 2011 with suspected FBA. We analyzed only patients who were exclusively admitted to the pediatric ward with a diagnosis of suspected FBA. Patients who underwent a bronchoscopy for other indications or required a bronchoscopy to exclude FBA later in the course of their illness, but were not admitted with a diagnosis of suspected FBA, were excluded. This study was approved by the research and ethics committee of the Hamad Medical Corporation (RC 11099/11).

### Study measures and data variables

The charts of the patients who met the eligibility criteria were retrospectively reviewed in detail. The history, presenting symptoms, clinical assessments as documented by the admitting pediatrician at the time were collected. Most of the patients (242, 80.7%) had an electronic record of the chest x-ray (CXR) that was read by the radiologist at the time of admission. These were noted and analyzed.

All 300 of these patients underwent at least one bronchoscopy, either flexible or rigid or both, depending upon their presentation. Generally, if there was a very definite history of witnessed choking and the patient presented with significant physical examination findings such as stridor, reduced air entry, hypoxia, tachypnea or obvious CXR abnormalities like radio-opaque FB, mediastinal shift, unilateral atelectasis/hyperinflation or a combination of any of these, the patient was referred directly to the pediatric surgical team who then proceeded for urgent rigid bronchoscopy under general anesthesia. If the patient presented with more subtle/less apparent radiological or physical examination findings, the pediatric pulmonology team was consulted to consider a flexible bronchoscopy as the initial procedure of choice. This trend of selecting rigid versus flexible bronchoscopy, based on the patients’ initial presentation, remained unchanged in the years pertaining to our study.

The bronchoscopy records were reviewed for details like type (flexible versus rigid), size of bronchoscope and instrumentation used for foreign body (FB) extraction, outcomes including presence, type and location of FB and pre- and post- procedure complications.

### Statistical analysis

The focus of the data analysis in our study was to determine the predictive accuracy of the various predictors and covariates in diagnosing FBA. For this, the sensitivity, specificity, positive and negative predictive values of these parameters were calculated, using bronchoscopy diagnosis of FBA as the point of reference. A receiver operating characteristic (ROC) curve was calculated using significant predictors (as determined via multivariate regression) to derive best suitable cut-off values and to assess model discrimination and predictive accuracy. ROC curves provide a comprehensive and visually attractive way to summarize the accuracy of predictions. The ROC curve shows the tradeoff between sensitivity and specificity and is a better method to detect the performance of a developed test, which classifies subjects into two categories such as FBA positive and FBA negative.

Associations between two or more qualitative variables were assessed using chi-square test and Fisher Exact test as appropriate. Univariate and multivariate logistic regression methods were used to assess the predictive values of each predictor or risk factors (clinical signs and symptoms, physical exam and radiological findings) for bronchoscopy proven FBA. For multivariate regression models, variables were considered if statistically significant at the *P* < 0.10 level in univariate analysis or if determined to be clinically important. The results of logistic regression analyses were reported as odds ratio (OR) with 95% confidence intervals (CIs). The number of risk factors identified in multivariable logistic regression analyses were summarized to compute weighted risk score and generate a clinically applicable and decision making rule for the prediction of FBA. To derive a simple-to-compute risk score, the regression coefficients were divided by the smallest coefficient and then rounded to the nearest integer. Pictorial presentations of the key results were made using appropriate statistical graphs. A two-sided *P* value <0.05 was considered to be statistically significant. All statistical analyses were done using statistical packages SPSS 22.0 (SPSS Inc. Chicago, IL) and Epi Info 2000 (Centers for Disease Control and Prevention, Atlanta, GA).

## Results

### Patient characteristics

Based on the inclusion criteria, a total of 300 children were included in this study. Of the 300 patients, 163 were boys (54.3%) and 137 were girls (45.7%), giving a male-to-female ratio of 1.2:1. A total of 139 children (46.3%) were Qatari nationals. The mean age was 2.1 ± 1.7 years (range 7 months to 14 years) with a peak incidence at 1 to 2 years of age. Seventy patients (23.3%) were less than 12 months of age.

These 300 patients cumulatively underwent a total of 410 bronchoscopies, which included both flexible and rigid bronchoscopies (88 patients underwent 2 bronchoscopies, 17 had 3, 4 patients had 4 and one patient underwent 5 bronchoscopies). Only 190 (66.7%) had a definite history of FBA. FB was found in the airway in 91 of the 300 children (30.3%). In all of these children, the FB was successfully extracted. The demographic characteristics, clinical, laboratory and bronchoscopy findings of the study population are shown in Table 1.

**Table 1:** Demographic characteristics, clinical, laboratory and bronchoscopy findings

Characteristics	N	Frequency (%)
Gender		
Male Female	300	163 (54.3) 137 (45.7)
Nationality		
Qatari Non-Qatari	300	139 (46.3) 161 (53.7)
Age (years)		
< 1 year 1 to <2 year 2 to < 3 year 3 to < 4 year 4 to <7 year 7–14 year	300	70 (23.3) 122 (40.7) 37 (12.3) 26 (8.7) 27 (9) 18 (6)
Bronchoscopy Episode		
One Two Three Four Five	410	300 (73.2) 88 (21.5) 17 (4.1) 4 (1) 1 (0.2)
Method of Bronchoscopy		
Flexible Rigid	410	318 (77.6) 92 (22.4)
History of Foreign Body^a^		
Definite Suspected	285	190 (66.7) 95 (33.3)
Foreign Body Aspiration		
Foreign Body Present No Foreign Body	300	91 (30.3) 209 (69.7)
Type of Foreign Body		
Organic Non-organic Type of FB not specified	91	57 (62.6) 8 (8.8) 26 (28.6)
Organic Foreign Body		
Nuts Seed Bone Others (not specified)	57	28 13 3 13
Inorganic Foreign Body		
Inorganic sharp object Inorganic blunt object Others (not specified)	8	5 2 1
Location and site of FB		
Right airways Left airways Trachea Others	91	43 (47.3) 32 (35.2) 6 (6.6) 10 (10.9)
Route of Bronchoscopy		
Nasally Orally Through ETT	410	306 (74.6) 93 (22.7) 11 (2.7)
Broncho-alvelolar lavage		
Yes No	300	56 (18.7) 244 (81.3)
Bronchoalveolar culture findings		
Negative Positive	56	36 (64.3) 20 (35.7)
Complications during procedure		
Transient hypoxia Laryngospasm Bronchospasm Nose bleed Bradycardia Intubation Others	16	8 (2) 1 (0.2) 1 (0.2) 1 (0.2) 3 (0.7) 1 (0.2) 1 (0.2)
Complications after procedure		
Excessive cough Pneumonia Stridor Tachypnea Tension Pneumothorax Others	8	3 (0.7) 1 (0.2) 1 (0.2) 1 (0.2) 1 (0.2) 1 (0.2)

*FBA* Foreign body aspiration

^a^In 15 cases no documentation was present in their medical records regarding the history of FB

### Clinical signs and symptoms

Positive history of noisy breathing/stridor/dysphonia and recurrent/persistent wheezing were notably more common in children with FBA compared to children without FBA. There was no significant difference observed in the occurrence of other clinical signs and symptoms between the two groups (*P* > 0.05) as shown in Table 2. In children with bronchoscopically proven FBA, the two most common presenting symptoms were; cough in 79.1% and witnessed choking in 71.4%. However, both witnessed choking and cough had a low specificity i.e. 36 and 31.1% respectively (Tables 3 and 4). Therefore, it can be concluded that the presence of either of these cannot be relied upon for an accurate diagnosis of FBA.

**Table 2:** Association of clinical sign and symptoms, physical and radiologic findings between children with recovery of a foreign body during bronchoscopy (FBA positive) and those without FBA (FBA negative) (univariate logistic regression analysis)

	FBA positive	FBA negative	Unadjusted odds ratio (95%CI)	*P*-value*
Clinical signs and symptoms (*n* = 300)				
Witnessed choking	65 (71.4%)	134 (64.1%)	1.39 (0.82, 2.39)	0.219
Cough	72 (79.1%)	144 (68.9%)	1.71 (0.95, 3.07)	0.072
Noisy breathing/stridor/ dysphonia	21 (23.1%)	23 (11%)	2.43 (1.26, 4.66)	0.008
Dyspnea	32 (35.2%)	57 (27.3%)	1.45 (0.85, 2.45)	0.170
Cyanosis	11 (12.1%)	46 (22%)	0.49 (0.24, 0.99)	0.047
New onset wheezing/ recurrent/persistent wheeze	20 (22%)	14 (6.7%)	3.92 (1.89, 8.18)	0.0001
Recurrent and/or persistent respiratory infection/ recurrent pneumonia	0 (0%)	2 (1%)	n.a	0.999
Vomiting	14 (15.4%)	35 (16.7%)	0.90 (0.46, 1.78)	0.769
Fever	21 (23.1%)	37 (17.7%)	1.40 (0.76, 2.55)	0.280
Others	8 (8.8%)	26 (12.4%)	0.68 (0.29, 1.56)	0.362
Radiological findings (*n* = 242)				
Abnormal	63 (82.9%)	87 (52.4%)	4.40 (2.25, 8.60)	0.0001
Unilateral hyperinflation right/left side	31 (40.8%)	34 (20.5%)	2.68 (1.48, 4.84)	0.001
Bilateral hyperinflation	10 (13.2%)	20 (12%)	1.11 (0.49, 2.49)	0.808
Right/left/bilateral infiltrates	10 (13.2%)	11 (6.6%)	2.14 (0.87, 5.27)	0.100
Radio-opaque FB	4 (5.3%)	0 (0%)		0.009
Collapse/Consolidation	6 (7.9%)	11 (6.6%)	1.21 (0.43, 3.40)	0.720
Others	2 (2.6%)	7 (4.2%)	0.61 (0.13, 3.03)	0.549
Physical examination findings (*n* = 290)				
Normal	21 (23.3%)	112 (56%)	0.24 (0.14, 0.42)	0.0001
Unilateral reduced entry Right/left side	53 (58.9%)	54 (27%)	3.87 (2.30, 6.54)	0.0001
Stridor	1 (1.1%)	3 (1.5%)	0.74 (0.08, 7.19)	0.794
Wheezing	29 (32.2%)	37 (18.5%)	2.09 (1.19, 3.70)	0.011
Bilateral reduced air entry	2 (2.2%)	11 (5%)	0.43 (0.09, 2.01)	0.285
Retractions	9 (10%)	15 (7.5%)	1.37 (0.58, 3.26)	0.476
Tachypnea	5 (5.6%)	3 (1.5%)	3.86 (0.90, 16.53)	0.068
Crackles	12 (13.3%)	21 (10.5%)	1.31 (0.62, 2.80)	0.483
Others	4 (4.4%)	3 (1.5%)	3.05 (0.67, 13.94)	0.149

In 58 cases Chest-X-ray was not done/and or not reported and hence % computation was based on *n* = 242

In 10 cases physical exam findings were not documented and thus % computation was based on *n* = 290

*OR* odds ratio, *CI* confidence interval

*Pearson Chi-square and Fisher exact test, logistic regression analysis

**Table 3:** Multivariate logistic regression model for determining significant predictors and deriving risk score for FBA

Predictor category	Predictor	FBA positive	FBA negative	Regression coefficient	Adjusted odds ratio^a^ (95%CI)	*P*-value*	Weight risk score^b^
Clinical sign and symptoms	Witnessed choking	65 (71.4%)	134 (64.1%)	0.753	2.12 (1.10, 4.38)	0.041	1
Clinical sign and symptoms	Noisy breathing/ Stridor/ Dysphonia	21 (23.1%)	23 (11%)	1.01	2.74 (1.22, 6.17)	0.015	1
Clinical sign and symptoms	New onset wheezing/ Recurrent/persistent wheeze	20 (22%)	14 (6.7%)	1.52	4.57 (1.77, 11.76)	0.002	2
Radiographic	Abnormal Chest X-ray findings	31 (40.8%)	34 (20.5%)	1.39	4.01 (1.88, 8.53)	<0.001	2
Physical examination	Unilateral reduced air entry	21 (23.3%)	112 (56%)	1.07	2.90 (1.53, 5.52)	0.001	1

*OR* odds ratio, *CI* confidence interval

*Pearson Chi-square and Fisher exact test

^a^Adjusted for all other potential covariates found in the univariate logistic regression analysis

^b^The scores were computed and derived by dividing the regression coefficients of the included predictors by the smallest regression coefficient and then rounding them to the nearest integer. For each patient, a sum score was calculated by adding the scores that correspond to the predictors and characteristics of the patient

**Table 4:** Diagnostic value of clinical sign and symptoms, radiological and physical exam findings with statistically significant predictors derived from logistic regression for proven foreign body aspiration

	Sensitivity (%)	Specificity (%)	PPV (%)	NPV (%)	*P*-value*
Noisy breathing/stridor/dysphonia	23.1	89.0	47.7	72.7	0.008
Cyanosis	12.1	78.0	19.3	67.1	0.047
New onset wheezing/recurrent/persistent wheeze	22	93.3	58.8	73.3	0.0001
Abnormal Chest-X-ray	82.9	47.6	42	85.9	0.0001
Unilateral hyperinflation right/left side	40.8	79.5	47.7	74.6	0.001
Radio-opaque FB	5.3	100	100	69.8	0.009
Normal physical examination	23.3	44.0	15.8	56.1	0.0001
Unilateral reduced air entry right/left side	32.2	81.5	43.9	72.8	0.011
Wheezing	58.9	73.0	49.5	79.8	0.0001

*PPV* positive predictive value, *NPV* negative predictive value

*Pearson Chi-square and Fisher exact test

### Physical exam findings

Decreased air entry (58.9% vs 27%; *P* = 0.0001) and wheezing (32.2% vs 18.5%; *P* = 0.011) were significantly more common in children with FBA compared to children without FBA. Children without FBA were also more likely to have a completely normal physical exam when compared to children with FBA (56% vs 23.3%; *P* = 0.0001). No statistically significant differences were observed in the frequencies of other related physical exam findings between children with FBA and those without FBA (*P* > 0.05); refer to Table 2.

### Chest Radiographic (CXR) findings

Of the 91 children with bronchoscopically proven FBA, 13 (17.1%) had normal chest radiographs, 31 (40.8%) and 10 (13.2%) had unilateral and bilateral hyperinflation, respectively. Ten patients had either right/left or bilateral lung infiltrates, six patients (7.9%) had collapse-consolidation, and 4 (5.3%) had radio-opaque FB and 2 had other findings. In 15 cases the CXR was either not done or not reported. Of note, children in the FBA group were significantly less likely to have a normal CXR compared to children without FBA (*P* < 0.0001). There were no significant differences observed in the occurrence of other radiological findings between the two groups (*P* > 0.05) as shown in Table 2, apart from the finding of a radio-opaque FB, which had a specificity and positive predictive value of 100% (Tables 3 and 4).

### Bronchoscopy findings

As stated previously, 91 (30.3%) of the 300 patients had a bronchoscopically confirmed diagnosis of FBA. In all of these cases, the FB was successfully removed; in 67 cases (74.4%) with rigid bronchoscopy and in 23 patients (25.6%) using a flexible bronchoscope. One patient required a thoracotomy. The flexible bronchoscopy was conducted by an experienced pediatric pulmonologist in most cases via the nasal route under moderate sedation in an intensive care setting. In 6 patients, it was performed in the operating theatre through an endotracheal tube under general anesthesia with a surgeon on standby. On the other hand, rigid bronchoscopies were performed by pediatric surgeons in the operating theatre under general anesthesia. It is of note that all but 6 cases that had their FB extracted via rigid bronchoscopy underwent a diagnostic flexible bronchoscopy by a pediatric pulmonologist before the rigid bronchoscopy was undertaken for FB extraction. Post bronchoscopy complications were reported in only 2.6% cases, with no procedure related mortalities (Table 1).

Of the 300 cases, 82 patients underwent multiple bronchoscopies. Twelve of these were patients who did not have a FB visualized during their initial procedure, but had a subsequent bronchoscopy performed a day or two later to exclude any missed FB as they remained symptomatic. The remaining 70 patients had a FB, but required multiple bronchoscopies due to various reasons. 45 patients had a flexible bronchoscopy performed for diagnostic purposes, and once the FB was located, a rigid bronchoscopy was done for FB extraction. In 8 patients, there was an unsuccessful attempt to extract the FB via flexible bronchoscopy, necessitating a second rigid bronchoscopy. In another 8 patients, the initial bronchoscopy (either rigid or flexible) extracted the FB incompletely; needing a second bronchoscopy for extracting the remnants. Nine patients were complicated cases who were admitted to the intensive care unit from the outset, they underwent multiple bronchoscopies including follow up bronchoscopies performed to rule out retained FB. One of these 9 patients eventually required a thoracotomy.

The most common site of FB lodging was the right main stem bronchus (47.3%), followed by the left bronchus (32, 35.2%), bilateral bronchi (5, 5.5%), carina (5, 5.5%) and the trachea (6, 6.6%). The rest were recovered from segmental smaller airways. Organic FB accounted for 62.6% of the FB removed, with peanuts (*n* = 15) being the most common type, followed by seeds (*n* = 13) and beans (*n* = 4), pistachios, and other food materials. Eight (8.8%) of the FB were inorganic; most of them were either metal or plastic objects, out of which 4, as stated earlier, were radio-opaque. In the remaining 28.6% cases, the patients’ records did not specify the type of FB. Table 1 outlines the bronchoscopy findings.

### Predictors associated with proven FBA and risk scoring system

**Fig. 1:**
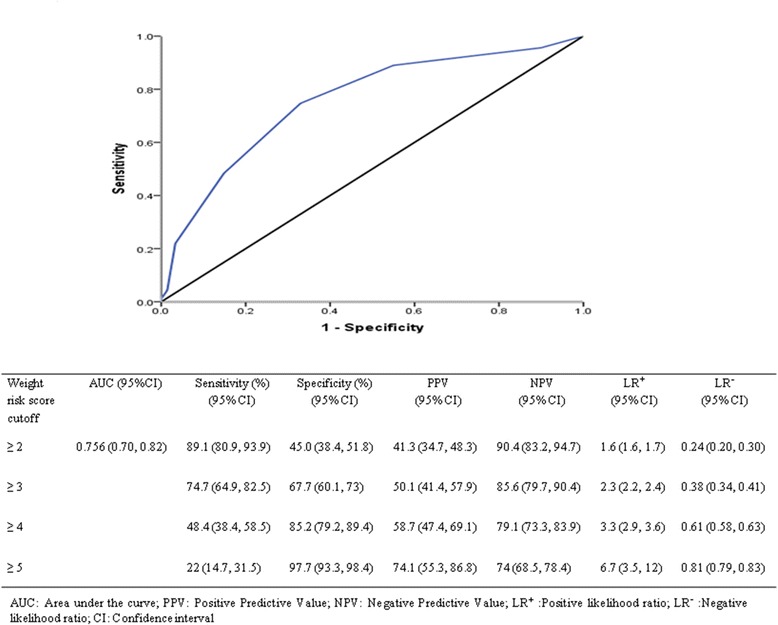
ROC curve using weighted risk score in predictive diagnosis of FBA

**Fig. 2:**
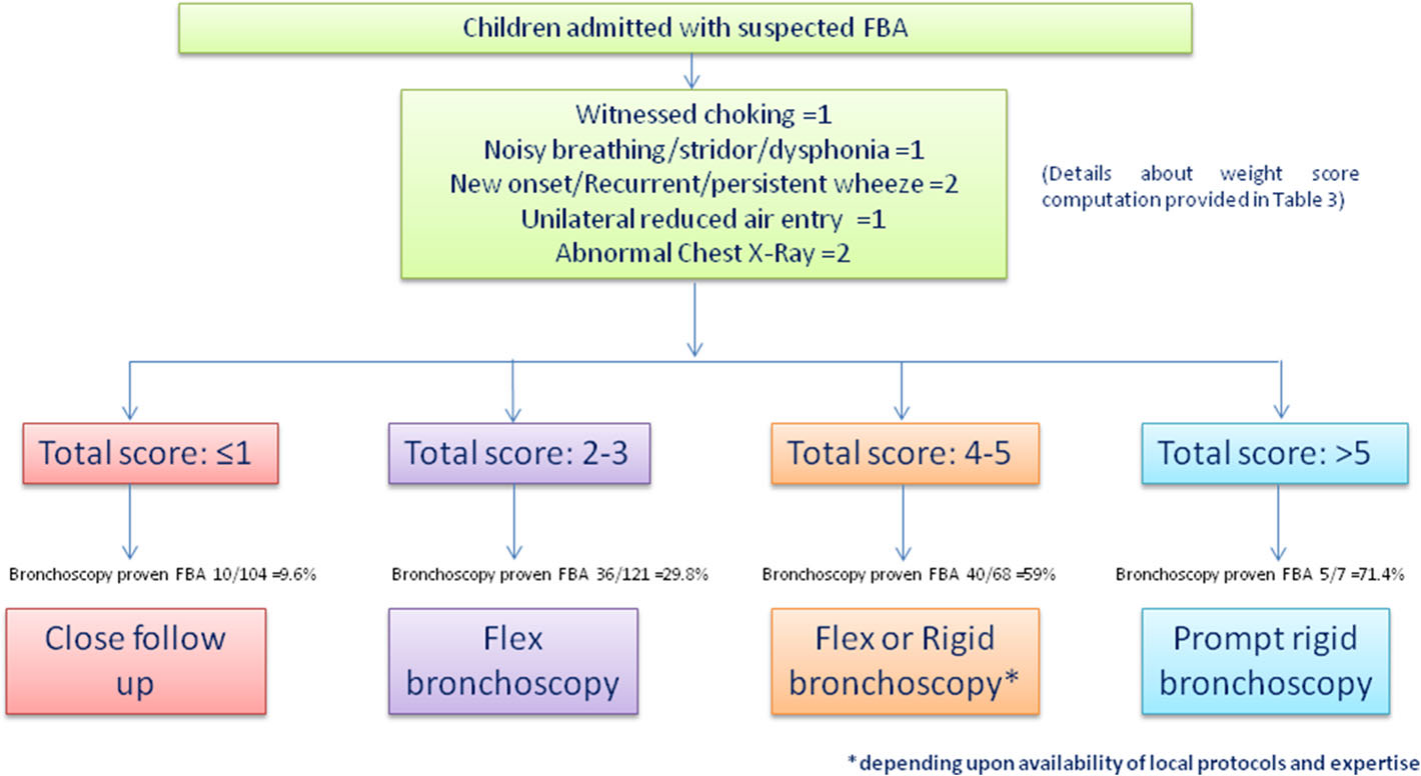
Clinical algorithm with scoring system for children with suspected FBA

Historical, clinical, physical exam and radiological findings associated with FBA are shown in Table 2. The derivation of the weighted risk score for FBA is shown in Table 3. The five predictors selected for the risk score were history of witnessed choking (adjusted OR 2.1, 95% CI 1.1–4.3; *P* = 0.041), noisy breathing/stridor/ dysphonia (adjusted OR = 2.7, 95% CI 1.2–6.2; *P* = 0.015), new onset wheezing/ recurrent/persistent wheeze (adjusted OR 4.6, 95% CI 1.8–11.8; *P* = 0.002), abnormal chest x-ray findings (adjusted OR 4.0, 95% CI 1.9–8.5; *P* < 0.001), and unilateral reduced air entry (adjusted OR 2.9, 95% CI 1.5–5.5; *P* = 0.001) were significant predictors associated with an increased risk for FBA (*P* < 0.05). The clinical feature of witnessed choking on history was used as the reference regression coefficient and assigned a weighted value of 1. The discriminative ability of the model was found to be good with an area under the ROC curve value of 0.76 (95% CI 0.70, 0.82). The sensitivity and specificity values at a cutoff score point of ≥4 were 49 and 85%, and at a cutoff score point of ≥5 were 22 and 98% (positive likelihood ratio 6.6), respectively (Fig. 1). The predicted cutoff score derived using ROC analysis was found to co-relate well with known clinically significant predictors of FBA (Fig. 2).

Based on the aforementioned results and statistically significant predictors associated with FBA derived from multivariate logistic regression, we designed a clinical algorithm with a scoring system. In our proposed algorithm, (Fig. 2), each of the significant FBA predictors namely witnessed choking, noisy breathing/stridor/dysphonia, new onset/recurrent/persistent wheeze, unilateral reduced air entry and abnormal CXR are assigned a weighted risk score of 1, 1, 2, 1 and 2 respectively. Patients with a score of 5 or more had nearly 72% bronchoscopy proven FBA. For a child with a score range of 4–5, our study demonstrated that 59% had bronchoscopy proven FBA. In patients with a score of 2–3, the bronchoscopy proven FBA was ~29.8%, while with a score of ≤1, the bronchoscopy proven FBA dropped to no more than 9.6%.

**Fig. 3:**
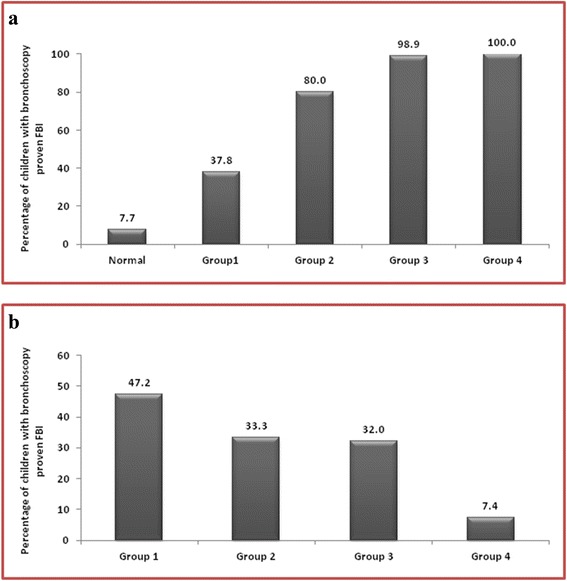
**a** Cumulative proportions of proven FBA by number of risk factors (witnessed choking crisis, noisy breathing/stridor/dysphonia, new onset wheezing/ recurrent/ persistent wheeze, and/or decreased air entry). Normal = patients with none of the above risk factors, Group 1 = patients with 1 risk factor, Group 2 = patients with any of the 2 above risk factors, Group 3 = patients with any of the 3 above risk factors and Group 4 = patient with all of the above risk factors. **b** Incidence of FBA found on bronchoscopy. Group categorizations: Group 1, children with abnormal physical and radiologic findings (*n* = 110); group 2, children with abnormal physical and normal radiologic findings (*n* = 30); group 3, children with normal physical and abnormal radiologic findings (*n* = 25); group 4, children with normal physical and radiological findings (*n* = 67). The sum is not equal 300 as in 58 cases either CXR was not done or not documented and therefore all the percentages were calculates based on 242 cases

Patients were also grouped based on the cumulative presence of 4 of the above 5 mentioned FBA predictors (witnessed choking crisis, Noisy breathing/stridor/dysphonia, New onset wheezing/recurrent/persistent wheeze, and/or decreased air entry). Figure 3a shows the cumulative proportion of children with proven FBA according to these 4 risk factors. Only 7.7% of the children without any of these risk factors had a proven FBA. The likelihood increased significantly with an increasing number of risk factors. When all 4 risk factors were present, the likelihood of FBA was 100%. A total of 242 of these 300 patients were also analyzed and grouped based on whether they had normal or abnormal physical and/or radiological findings on presentation. The remainder of the patients did not have a documented exam and/or chest radiological findings on record review. The results were as follows: (Fig. 3b) in children with both abnormal physical and radiological findings, 47.2% had a proven FBA. If only one was abnormal (i.e. either physical exam or chest x-ray), the likelihood of FBA reduced to 32–33.3%. In children who presented with a completely normal physical exam and chest radiograph, only 7.4% had a FB removed by bronchoscopy (Fig. 3b).

## Discussion

Children with suspected FBA will always present a perplexing diagnostic dilemma because the condition can only be established unequivocally after the patient has undergone a bronchoscopy [11]. There is no global consensus regarding reliable indications for bronchoscopy in children with suspected FBA. This variation in practice is also reflected in the different rates of positive bronchoscopies, among different centers around the world. In our study, about one third of the patients (30.3%) who underwent bronchoscopy for suspected FBA had positive findings. In a similar study by Shlomo Cohen et al. [5] the rate of positive bronchoscopies was 45%. Other studies from around the world quote positive bronchoscopy rates, which vary widely, ranging from as low as 25 to over 90% [12–16].

There is not only a significant discrepancy among institutions, regarding the management of these children, but frequently also among physicians working in the same center. The decision is often based wholly on the discretion of the treating physician. Many previous studies have attempted to establish the criteria for bronchoscopy in children with FBA by drawing conclusions from the presenting history, examination and chest radiograph findings of patients with bronchoscopically proven FBA [2, 5, 17–20].

In this study, we attempted to go a step further by utilizing the statistically significant predictors we identified based on cases with bronchoscopically proven FBA to formulate a clinical algorithm with a scoring system which would help to decide which sub set of patients who present with a history suggestive of FBA require a prompt bronchoscopy. Using multivariable logistic regression analysis controlling for all other potential predictors, we found that the predictors with the strongest association with FBA are witnessed choking crisis, noisy breathing/stridor/dysphonia, new onset, recurrent or persistent wheeze, abnormal chest x-ray findings, and unilateral reduced air entry (Tables 3 and 4). No significant interactions were observed between the various other historical signs and symptoms, radiological and physical examination findings. The discriminative ability of the model was found to be good with an area under the ROC curve value of 0.76 (95% CI 0.70, 0.82). The predicted cutoff score derived using statistically significant predictors and ROC analysis was found to be almost similar to that derived from clinically significant predictors and supports the clinical algorithm and scoring shown in Fig. 2.

Figure 3a demonstrates the cumulative effect of 4 of the predictive risk factors (witnessed choking crisis, noisy breathing/stridor/dysphonia, new onset wheezing/ recurrent/persistent wheeze, and/or decreased air entry) derived from our study, with the probability of finding a FB increasing to 100% when all 4 risk factors were present. Heyer CM et al. [21] reported cumulative proportions of children with proven FBA according to three risk factors, focal hyperinflation, witnessed choking crisis, and elevated white blood cell count and observed that sixteen percent of the children without any of these risk factors still had proven FBA, but the probability of proven FBA increased sharply with an increasing number of risk factors, just like in our study as seen in Fig. 3a.

It was noted that among patients who had both a pathological physical exam finding and an abnormal chest radiograph nearly half the cases (47.2%) had a FB on bronchoscopy. This is in contrast to children who presented with a completely normal physical exam and chest radiograph, where only 7.4% had evidence of FBA (Fig. 3b). Shlomo et al. had similar findings and concluded that patients with no physical or radiological abnormalities did not require a bronchoscopy as long as they were asymptomatic [5].

Based on our novel algorithm (Fig. 2), patients with a score of 5 or more had nearly 72% bronchoscopy proven FBA. We recommend performing a prompt rigid bronchoscopy in these cases, as the risk of FBA is quite high [5, 17]. For a patient with a score range of 4–5, with 59% bronchoscopy proven FBA, we propose either a flexible or rigid bronchoscopy based on the availability of local protocols and expertise of individual centers. In patients with a score of 2–3, where the bronchoscopy proven FBA was ~29.8%, the procedure of choice would be a flexible bronchoscopy to avoid general anesthesia in the large number of patients who may not have a FB. When the score is less than or equal to 1 on our algorithm, patients can be safely followed-up on an outpatient basis as long as close monitoring is available since the bronchoscopy proven FBA was found to be just under 10%.

In our study, based on Fig. 2, there were 4 patients who were eventually diagnosed with a FB but did not fulfill the predictive criteria as per our algorithm, i.e. had a score of “0”. Two of these four cases were toddlers who had chronic upper respiratory tract symptoms including persistent cough. Another presented with radiological evidence of right lower lobe bronchiectasis and the fourth with a unilateral foul smelling purulent nasal discharge with a FB extracted from the left nasal passage. In all except one of the aforementioned cases, the FB was successfully extracted via flexible bronchoscopy.

The major limitation of our study is that our data was collected and analyzed retrospectively and thus complete documentation regarding key clinical parameters such as history, CXR or physical exam findings were not available for all patients. On the other hand, the fact that this is a cohort data involving a large number of patients over a 10 year period makes this a valuable study.

## Conclusions

Accurately diagnosing FBA in children who need quick intervention will always be challenging. A high index of suspicion is required, along with a comprehensive historical account, detailed physical exam and chest radiography. Our proposed clinical algorithm and scoring system hopes to empower physicians dealing with such cases to accurately predict patients with a high likelihood of FBA. Although, the ultimate decision for bronchoscopy may be eventually based on the physician’s clinical judgment, having a reliable, statistically proven tool to base that decision on will be invaluable in initiating prompt management in those deemed high risk for FBA, as well as avoiding unnecessary intervention in those who have a very low probability of aspiration.

## Abbreviations

CI: Confidence intervals
CXR: Chest x-ray
FB: Foreign body
FBA: Foreign Body Aspiration
OR: Odds ratio
ROC: Receiver operating characteristic
